# Supplementary material for: Analysis of Mycotoxin and Secondary Metabolites in Commercial and Traditional Slovak Cheese Samples
Source: Toxins (Basel). 2022 Feb 10;14(2):134. doi: 10.3390/toxins14020134 (PMC8878695; doi:10.3390/toxins14020134)
Supplement: Supplementary file 1 [file toxins-14-00134-s001.zip › toxins-1595925-supplementary.pdf]

toxins

Supplementary Materials

Analysis of Mycotoxin and Secondary Metabolites in Commercial and Traditional Slovak Cheese Samples

Luana Izzo, Petra Mikušová, Sonia Lombardi, Michael Sulyok and Alberto Ritieni

Table S1. Occurrence of 13 target compounds in Slovak cheese samples (n = 68). Results are expressed as µg/kg samples.

Sample	3-NPA*	Andrastin A	Andrastin B	Andrastin C	Andrastin D	Chanoclavine	Enniatin B	Festuclavine	iso-Fumigaclavine	Mycophenolic acid	Roquefortine C	Roquefortine D	Tryptophol
							µg/kg						
Traditional Slovak cheeses:													
1	<LOD	<LOD	<LOD	<LOD	<LOD	<LOD	0.51	<LOD	<LOD	<LOD	<LOD	<LOD	41.2
2	<LOD	<LOD	<LOD	<LOD	<LOD	<LOD	0.36	<LOD	<LOD	<LOD	<LOD	<LOD	<LOQ
3	<LOD	<LOD	<LOD	<LOD	<LOD	<LOD	0.22	<LOD	<LOD	<LOD	<LOD	<LOD	7930
4	66.7	8890	3610	3150	115.7	3.1	0.39	1.2	178	29.4	8055	591.8	<LOQ
5	<LOD	<LOD	<LOD	<LOD	<LOD	<LOD	0.71	<LOD	<LOD	<LOD	<LOD	<LOD	162.7
6	10.6	7510	2750	3570	89.7	7.5	0.26	2	294	20.7	17900	924	298.5
7	<LOD	<LOD	<LOD	<LOD	<LOD	<LOD	0.45	<LOD	<LOD	<LOD	<LOD	<LOD	<LOD
8	<LOD	<LOD	<LOD	<LOD	<LOD	<LOD	0.07	<LOD	<LOD	<LOD	<LOD	<LOD	<LOD
9	<LOD	<LOD	<LOD	<LOD	<LOD	<LOD	0.10	<LOD	<LOD	<LOD	<LOD	<LOD	<LOD
10	<LOD	<LOD	<LOD	<LOD	<LOD	<LOD	0.06	<LOD	<LOD	<LOD	<LOD	<LOD	<LOD
11	<LOD	<LOD	<LOD	<LOD	<LOD	<LOD	0.04	<LOD	<LOD	<LOD	<LOD	<LOD	<LOD
12	<LOD	<LOD	<LOD	<LOD	<LOD	<LOD	0.04	<LOD	<LOD	<LOD	<LOD	<LOD	<LOD
13	<LOD	<LOD	<LOD	<LOD	<LOD	<LOD	0.16	<LOD	<LOD	<LOD	<LOD	<LOD	13.4
14	<LOD	<LOD	<LOD	<LOD	<LOD	<LOD	0.11	<LOD	<LOD	<LOD	<LOD	<LOD	<LOD
15	<LOD	<LOD	<LOD	<LOD	<LOD	<LOD	0.04	<LOD	<LOD	<LOD	<LOD	<LOD	<LOD
16	<LOD	<LOD	<LOD	<LOD	<LOD	<LOD	0.69	<LOD	<LOD	<LOD	<LOD	<LOD	241.1
17	<LOD	<LOD	<LOD	<LOD	<LOD	<LOD	0.22	<LOD	<LOD	<LOD	<LOD	<LOD	374.1
18	<LOD	<LOD	<LOD	<LOD	<LOD	<LOD	0.07	<LOD	<LOD	<LOD	<LOD	<LOD	15.00
19	<LOD	<LOD	<LOD	<LOD	<LOD	<LOD	0.17	<LOD	<LOD	<LOD	<LOD	<LOD	30.7
20	<LOD	<LOD	<LOD	<LOD	<LOD	<LOD	0.29	<LOD	<LOD	<LOD	<LOD	<LOD	<LOD
21	<LOD	<LOD	<LOD	<LOD	<LOD	<LOD	0.11	<LOD	<LOD	<LOD	<LOD	<LOD	<LOD
22	<LOD	<LOD	<LOD	<LOD	<LOD	<LOD	0.04	<LOD	<LOD	<LOD	<LOD	<LOD	<LOD
23	<LOD	<LOD	<LOD	<LOD	<LOD	<LOD	0.17	<LOD	<LOD	<LOD	<LOD	<LOD	<LOD
24	<LOD	<LOD	<LOD	<LOD	<LOD	<LOD	0.06	<LOD	<LOD	<LOD	<LOD	<LOD	<LOD
25	<LOD	<LOD	<LOD	<LOD	<LOD	<LOD	0.12	<LOD	<LOD	<LOD	<LOD	<LOD	<LOD

toxins

26	<LOD	<LOD	<LOD	<LOD	<LOD	<LOD	0.29	<LOD	<LOD	<LOD	<LOD	<LOD	52.6
27	<LOD	<LOD	<LOD	<LOD	<LOD	<LOD	0.31	<LOD	<LOD	<LOD	<LOD	<LOD	<LOD
28	<LOD	<LOD	<LOD	<LOD	<LOD	<LOD	0.08	<LOD	<LOD	<LOD	<LOD	<LOD	<LOD
29	<LOD	<LOD	<LOD	<LOD	<LOD	<LOD	0.09	<LOD	<LOD	<LOD	<LOD	<LOD	<LOD
Commercial Slovak cheeses:													
30	16.9	9140	1954	1579.5	152.3	6.9	0.53	0.21	90	16.2	13700	901.7	< LOD
31	3.8	< LOD	< LOD	< LOD	< LOD	< LOD	0.34	<LOD	<LOD	<LOD	<LOD	<LOD	171
32	<LOD	<LOD	<LOD	<LOD	<LOD	<LOD	0.11	<LOD	<LOD	<LOD	<LOD	<LOD	138.6
33	<LOD	<LOD	<LOD	<LOD	<LOD	<LOD	0.23	<LOD	<LOD	<LOD	<LOD	<LOD	<LOQ
34	<LOD	<LOD	<LOD	<LOD	<LOD	<LOD	0.13	<LOD	<LOD	<LOD	<LOD	<LOD	19.5
35	<LOD	<LOD	<LOD	<LOD	<LOD	<LOD	0.18	<LOD	<LOD	<LOD	<LOD	<LOD	17.4
36	<LOD	<LOD	<LOD	<LOD	<LOD	<LOD	0.07	<LOD	<LOD	<LOD	<LOD	<LOD	<LOD
37	27.2	4782	2448	3083	96.2	4.9	0.57	1.3	135.41	28.69	11853	679	<LOD
38	<LOD	<LOD	<LOD	<LOD	<LOD	<LOD	0.05	<LOD	<LOD	<LOD	<LOD	<LOD	<LOD
39	<LOD	<LOD	<LOD	<LOD	<LOD	<LOD	0.07	<LOD	<LOD	<LOD	<LOD	<LOD	<LOD
40	<LOD	<LOD	<LOD	<LOD	<LOD	<LOD	0.17	<LOD	<LOD	<LOD	<LOD	<LOD	<LOD
41	<LOD	<LOD	<LOD	<LOD	<LOD	<LOD	0.26	<LOD	<LOD	<LOD	<LOD	<LOD	<LOD
42	<LOD	<LOD	<LOD	<LOD	<LOD	<LOD	0.02	<LOD	<LOD	<LOD	<LOD	<LOD	<LOD
43	<LOD	<LOD	<LOD	<LOD	<LOD	<LOD	0.09	<LOD	<LOD	<LOD	<LOD	<LOD	304.5
44	<LOD	<LOD	<LOD	<LOD	<LOD	<LOD	0.08	<LOD	<LOD	<LOD	<LOD	<LOD	266.5
45	<LOD	<LOD	<LOD	<LOD	<LOD	<LOD	0.29	<LOD	<LOD	<LOD	<LOD	<LOD	17.3
46	<LOD	<LOD	<LOD	<LOD	<LOD	<LOD	0.08	<LOD	<LOD	<LOD	<LOD	<LOD	15.5
47	<LOD	<LOD	<LOD	<LOD	<LOD	<LOD	0.04	<LOD	<LOD	<LOD	<LOD	<LOD	99.2
48	<LOD	<LOD	<LOD	<LOD	<LOD	<LOD	0.11	<LOD	<LOD	<LOD	<LOD	<LOD	<LOD
49	<LOD	<LOD	<LOD	<LOD	<LOD	<LOD	0.19	<LOD	<LOD	<LOD	<LOD	<LOD	<LOD
50	<LOD	<LOD	<LOD	<LOD	<LOD	<LOD	0.16	<LOD	<LOD	<LOD	<LOD	<LOD	<LOD
51	<LOD	<LOD	<LOD	<LOD	<LOD	<LOD	0.17	<LOD	<LOD	<LOD	<LOD	<LOD	<LOD
52	<LOD	<LOD	<LOD	<LOD	<LOD	<LOD	0.17	<LOD	<LOD	<LOD	<LOD	<LOD	<LOQ
53	<LOD	<LOD	<LOD	<LOD	<LOD	<LOD	0.06	<LOD	<LOD	<LOD	<LOD	<LOD	151
54	<LOD	<LOD	<LOD	<LOD	<LOD	<LOD	0.26	<LOD	<LOD	<LOD	<LOD	<LOD	353.7
55	<LOD	<LOD	<LOD	<LOD	<LOD	<LOD	0.05	<LOD	<LOD	<LOD	<LOD	<LOD	<LOD
56	<LOD	<LOD	<LOD	<LOD	<LOD	<LOD	0.10	<LOD	<LOD	<LOD	<LOD	<LOD	<LOD
57	<LOD	<LOD	<LOD	<LOD	<LOD	<LOD	0.08	<LOD	<LOD	<LOD	<LOD	<LOD	<LOD
58	<LOD	<LOD	<LOD	<LOD	<LOD	<LOD	0.24	<LOD	<LOD	<LOD	<LOD	<LOD	<LOD
59	<LOD	<LOD	<LOD	<LOD	<LOD	<LOD	0.07	<LOD	<LOD	<LOD	<LOD	<LOD	<LOD
60	<LOD	<LOD	<LOD	<LOD	<LOD	<LOD	0.10	<LOD	<LOD	<LOD	<LOD	<LOD	<LOQ
61	<LOD	<LOD	<LOD	<LOD	<LOD	<LOD	0.10	<LOD	<LOD	<LOD	<LOD	<LOD	<LOQ
62	<LOD	<LOD	<LOD	<LOD	<LOD	<LOD	0.10	<LOD	<LOD	<LOD	<LOD	<LOD	72.32
63	<LOD	<LOD	<LOD	<LOD	<LOD	<LOD	0.20	<LOD	<LOD	<LOD	<LOD	<LOD	<LOD

toxins

64	<LOD	<LOD	<LOD	<LOD	<LOD	<LOD	0.07	<LOD	<LOD	<LOD	<LOD	<LOD	<LOD
65	<LOD	<LOD	<LOD	<LOD	<LOD	<LOD	0.17	<LOD	<LOD	<LOD	<LOD	<LOD	<LOD
66	<LOD	<LOD	<LOD	<LOD	<LOD	<LOD	0.11	<LOD	<LOD	<LOD	<LOD	<LOD	<LOD
67	<LOD	<LOD	<LOD	<LOD	<LOD	<LOD	0.09	<LOD	<LOD	<LOD	<LOD	<LOD	<LOD
68	<LOD	<LOD	<LOD	<LOD	<LOD	<LOD	0.13	<LOD	<LOD	<LOD	<LOD	<LOD	<LOD
LOD (µg/kg)	0.74	0.25	0.87	0.25	0.25	0.18	0.01	0.03	0.12	1.16	0.47	0.54	3.52
LOQ (µg/kg)	2.71	0.83	2.91	0.83	0.83	0.61	0.04	0.10	0.41	3.88	1.56	1.81	11.70

*3-Nitropropionic acid

Table S2. Characteristics of the 68 analyzed samples, original traditional Slovak cheeses came from markets and local farmers using a traditional biotechnical process at manufacture (n = 29) and common commercial Slovak cheeses came from markets (n = 39).

Sample ID	Type of Cheese	Commercial Name/Origin of Cheese
Traditional Slovak cheeses:		
1	Camembert (P. Candidum)/ Cow's Cheese [1]	-
2	Camembert (P. Candidum)/ Cow's Cheese [2]	-
3	Camembert (P. Candidum)/ Cow's Cheese [3]	-
4	Camembert (P. Candidum)/ Cow's Cheese	-
5	Camembert (P. Candidum)/ Cow's Cheese	-
6	Niva (P. Roquefortii) / Cow's Cheese	-
7	Niva (P. Roquefortii) / Cow's Cheese	-
8	Niva (P. Roquefortii) / Cow's Cheese	-
9	Niva (P. Roquefortii) / Cow's Cheese	-
10	Bryndza/ Sheep cheese	-
11	Bryndza/ Sheep cheese	-
12	Bryndza/ Sheep cheese	-
13	Bryndza/ Sheep cheese	-
14	Bryndza/ Sheep cheese	-
15	Bryndza/ Sheep cheese	-
16	Parenica/ Cow's Cheese	-
17	Parenica/ Cow's Cheese	-
18	Oštiepok/ Cow's Cheese	-

19	Baranec/Sheep Cheese	-
20	Nite/Cow's Cheese	-
21	Nite/Cow's Cheese	-
22	Nite/Cow's Cheese	-
23	Nite/Cow'S Cheese	-
24	Korbáčik/Cow's Cheese	-
25	Korbáčik/Cow's Cheese	-
26	Korbáčik/Cow's Cheese	-
27	Korbáčik/Cow's Cheese	-
28	Sheep Cheese Not Smoked	-
29	Sheep Cheese Smoked	-
Commercial Slovak cheeses:		
30	Sheep/Not Smoked/Lump	Ovčia Kolibka Spiš, Kluknavská Mliekáreň
31	Sheep/ Not Smoked/Lump	Čierny Balog
32	Sheep/ Not Smoked/Lump	Plachtická Farma Baránek, Dolné Plachtince
33	Sheep/ Not Smoked/Fresh	Plachtická Farma Baránek, Dolné Plachtince
34	Sheep/Not Smoked/Lump	Kochanovce
35	Sheep/Not Smoked/Lump	Kluknavská Mliekáreň, Jaklovce
36	Sheep/Not Smoked/Lump	Kozí Vŕšok, Ivachnová, Liptovská Teplá
37	Sheep/Smoked/Lump	Ovčia Kolibka Spiš, Kluknavská Mliekáreň
38	Sheep/Smoked/Lump	Agronova, Liptov, Farma Bukovina
39	Sheep/Smoked/Lump	Agronova, Liptov, Farma Bukovina
40	Cow/Semi-Soft, Ripening, Full-Fat/Lump	Agrofarma, Červený Kameň
41	Cow/Hard, Ripening, Full-Fat, /Lump	Agrofarma, Červený Kameň
42	Cow/Semi - Soft, Ripening Full - Fat/Lump	Syráreň Bel, Michalovce
43	Cow/Steamed, Semi-Hard, Ripening, Semi-Fat	Agrofarma, Červený Kameň
44	Cow/Steamed, Semi-Hard, Unripened, Semi-Fat	Agrofarma, Červený Kameň
45	Cow/Natural, Full-Fat, Unripened, Soft	Važec
46	Cow's Cheese	-
47	Cow/Fresh, Natural, Soft, Unripened, Full-Fat	Mliekáreň Sky Group/Považský Inovec. Stará Lehota
48	Cow/Soft, White	Moja Kravička/Slatina Nad Bebravou
49	Cow/Natural Ripening Semi - Hard, Semi - Fat	Syrošík, Farma Východná

toxins

50	Cow/Unripened, Full-Fat	Mliekáreň Sky Group/Považský Inovec. Stará Lehota
51	Cow/Semi-Hard Ripening Full-Fat	Syráreň Havran, Senica
52	Cow/Natural, Semi-Soft, Full-Fat	Volovec, Milk-Agro, Prešov
53	Cow/Ripening, Semi-Soft, Full-Fat	Liptov, Savencia Fromage and Dairy
54	Cow/Ripening, Semi-Soft, Full-Fat	Syráreň Bel, Michalovce
55	Cow/Ripening, Semi-Soft, Full-Fat	Levické Mliekárne, Levice
56	Cow/Ripening, Semi-Soft, Full-Fat	Koliba, Hriňová
57	Cow/Ripening, Semi-Soft, Full-Fat	Levické Mliekárne, Levice
58	Cow/Ripening, Semi-Soft, Full-Fat	Farma Baránek, Dolné Plachtince
59	Cow/Ripening, Semi-Soft, Full-Fat	Nika, Považská Bystrica
60	Cow/Ripening, Semi-Soft, Semi-Fat	Syráreň Bel, Michalovce
61	Cow/Ripening, Semi-Soft, Full-Fat	Milk-Agro, Čapajevova, Prešov
62	Cow/Ripening, Semi-Soft, Full-Fat	Koliba, Krivec, Hriňová
63	Cow/Ripening, Semi-Soft, Full-Fat	Syráreň Bel, Michalovce
64	Cow/Ripening, Semi-Soft, Full-Fat	Liptov, Savencia Fromage and Dairy
65	Goat/Fresh/Full-Fat	Plachtická Farma Baránek, Dolné Plachtince
66	Goat/Ripening/Full-Fat	Plachtická Farma Baránek, Dolné Plachtince
67	Goat/Ripening/Full-Fat	Leonteus, Ivachnová, Liptovská Teplá
68	Goat/Fresh/Full-Fat	Syrex, Zázrivá

***Commercial Name/Origin of The Cheese**: 1 Plesnivec, Tami, Tatranská Mliekáreň, 2 Encián, Tami, Tatranská Mlikáreň, 3 Encián With Olives, Tami, Tatranská Mliekáreň
